# Disruption of retinal inflammation and the development of diabetic retinopathy in mice by a CD40-derived peptide or mutation of CD40 in Müller cells

**DOI:** 10.1007/s00125-022-05775-6

**Published:** 2022-08-03

**Authors:** Jose-Andres C. Portillo, Jin-Sang Yu, Sarah Vos, Reena Bapputty, Yalitza Lopez Corcino, Alyssa Hubal, Jad Daw, Sahil Arora, Wenyu Sun, Zheng-Rong Lu, Carlos S. Subauste

**Affiliations:** 1 Division of Infectious Diseases and HIV Medicine, Department of Medicine, Case Western Reserve University, Cleveland, OH USA; 2 Department of Pediatrics, Case Western Reserve University, Cleveland, OH USA; 3 Department of Pathology, Case Western Reserve University, Cleveland, OH USA; 4 Department of Biomedical Engineering, Case Western Reserve University, Cleveland, OH USA

**Keywords:** CD40, Diabetic retinopathy, Endothelial cells, Inflammation, Microglia/macrophages, Müller cells

## Abstract

**Aims/hypothesis:**

CD40 expressed in Müller cells is a central driver of diabetic retinopathy. CD40 causes phospholipase Cγ1 (PLCγ1)-dependent ATP release in Müller cells followed by purinergic receptor (P2X_7_)-dependent production of proinflammatory cytokines in myeloid cells. In the diabetic retina, CD40 and P2X_7_ upregulate a broad range of inflammatory molecules that promote development of diabetic retinopathy. The molecular event downstream of CD40 that activates the PLCγ1–ATP–P2X_7_–proinflammatory cytokine cascade and promotes development of diabetic retinopathy is unknown. We hypothesise that disruption of the CD40-driven molecular events that trigger this cascade prevents/treats diabetic retinopathy in mice.

**Methods:**

B6 and transgenic mice with Müller cell-restricted expression of wild-type (WT) CD40 or CD40 with mutations in TNF receptor-associated factor (TRAF) binding sites were made diabetic using streptozotocin. Leucostasis was assessed using FITC-conjugated concanavalin A. Histopathology was examined in the retinal vasculature. Expression of inflammatory molecules and phospho-Tyr783 PLCγ1 (p-PLCγ1) were assessed using real-time PCR, immunoblot and/or immunohistochemistry. Release of ATP and cytokines were measured by ATP bioluminescence and ELISA, respectively.

**Results:**

Human Müller cells with CD40 ΔT2,3 (lacks TRAF2,3 binding sites) were unable to phosphorylate PLCγ1 and release ATP in response to CD40 ligation, and could not induce TNF-α/IL-1β secretion in bystander myeloid cells. CD40–TRAF signalling acted via Src to induce PLCγ1 phosphorylation. Diabetic mice in which WT CD40 in Müller cells was replaced by CD40 ΔT2,3 failed to exhibit phosphorylation of PLCγ1 in these cells and upregulate P2X_7_ and TNF-α in microglia/macrophages. *P2x*_*7*_ (also known as *P2rx7*), *Tnf-α* (also known as *Tnf*), *Il-1β* (also known as *Il1b*), *Nos2*, *Icam-1* (also known as *Icam1*) and *Ccl2* mRNA were not increased in these mice and the mice did not develop retinal leucostasis and capillary degeneration. Diabetic B6 mice treated intravitreally with a cell-permeable peptide that disrupts CD40–TRAF2,3 signalling did not exhibit either upregulation of P2X_7_ and inflammatory molecules in the retina or leucostasis.

**Conclusions/interpretation:**

CD40–TRAF2,3 signalling activated the CD40–PLCγ1–ATP–P2X_7_–proinflammatory cytokine pathway. Src functioned as a link between CD40–TRAF2,3 and PLCγ1. Replacing WT CD40 with CD40 ΔT2,3 impaired activation of PLCγ1 in Müller cells, upregulation of P2X_7_ in microglia/macrophages, upregulation of a broad range of inflammatory molecules in the diabetic retina and the development of diabetic retinopathy. Administration of a peptide that disrupts CD40–TRAF2,3 signalling reduced retinal expression of inflammatory molecules and reduced leucostasis in diabetic mice, supporting the therapeutic potential of pharmacological inhibition of CD40–TRAF2,3 in diabetic retinopathy.

**Graphical abstract:**

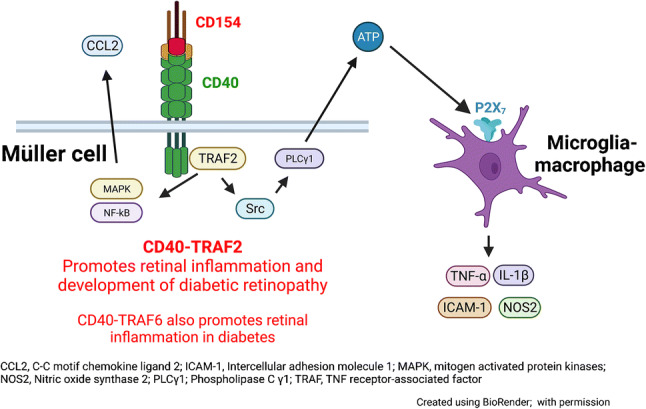

**Supplementary Information:**

The online version of this article (10.1007/s00125-022-05775-6) contains peer-reviewed but unedited supplementary material.

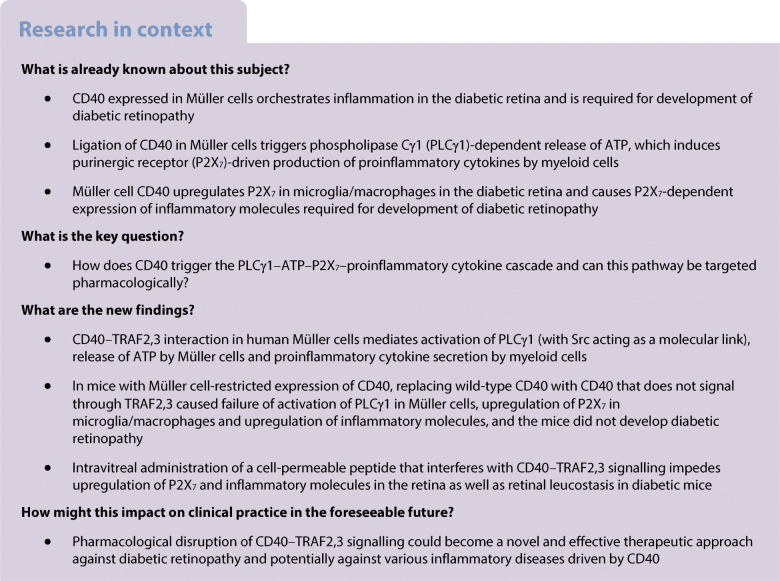



## Introduction

CD40 and its ligand CD154 are therapeutic targets against inflammatory diseases [1]. The importance of CD40 has been examined in animal models of diabetic retinopathy. These models contributed to understanding the pathogenesis of this disease since events that lead to early diabetic retinopathy are similar in humans and rodents [2, 3]. Studies in mice uncovered that CD40 induces inflammation in the diabetic retina and is required for the early development of diabetic retinopathy [4]. CD40 expression is increased in Müller cells, endothelial cells and microglia in the retina of diabetic mice [4]. Importantly, diabetic *Cd40*^−/−^ mice are protected from retinal upregulation of intercellular adhesion molecule 1 (ICAM-1), TNF-α, IL-1β, nitric oxide synthase 2 (NOS2) and C-C motif chemokine ligand 2 (CCL2) and do not develop diabetic retinopathy [4, 5]. These findings are consistent with evidence that low-grade chronic inflammation plays an important role in the development of diabetic retinopathy [6–8]. Moreover, an increase in leucocytes adhering to retinal vasculature (leucostasis) is linked to the development of capillary degeneration, a key feature of early diabetic retinopathy [9].

Studies in diabetic transgenic mice that expressed CD40 only in Müller cells demonstrated that the presence of CD40 in these cells is sufficient for upregulation of *Icam-1* (also known as *Icam1*), *Tnf-α* (also known as *Tnf*), *Il-1β* (also known as *Il1b*), *Nos2* and *Ccl2* mRNA as well as for the development of diabetic retinopathy characterised by capillary degeneration [5]. Interestingly, while CD40 ligation induces some proinflammatory responses in Müller cells (e.g. CCL2), CD40 stimulation does not result in TNF-α or IL-1β production by these cells [5]. Instead, CD40 ligation activates phospholipase Cγ1 (PLCγ1), causing Müller cells to release extracellular ATP. In turn, ATP engages the purinergic receptor P2X_7_ in myeloid cells resulting in secretion of TNF-α and IL-1β by these cells [5]. This mechanism enables Müller cells to trigger proinflammatory cytokine production in myeloid cells [5]. Moreover, the presence of CD40 in Müller cells in the retinas of diabetic mice results in TNF-α expression in retinal microglia/macrophages (but not in Müller cells), upregulation of P2X_7_ in microglia/macrophages and P2X_7_-dependent upregulation of TNF-α, IL-1β, NOS2 and ICAM-1 [5]. Thus, the pathway of Müller cell CD40–PLCγ1–ATP–P2X_7_ expressed on microglia/macrophages triggers proinflammatory cytokine production amplifying inflammation in the diabetic retina.

The role of CD40 as regulator of inflammation and development of diabetic retinopathy indicates that CD40 may represent a therapeutic target against this disease. Generalised disruption of the CD40–CD154 pathway was attempted in clinical trials of individuals with lupus nephritis and inflammatory bowel disease (CD40-driven diseases) using neutralising monoclonal antibody (mAb) against CD154 [10]. While this approach appeared to reduce disease activity, it induced thrombotic events unrelated to CD40 inhibition caused by binding of anti-CD154 mAbs to platelets [10]. In addition, global inhibition of CD40 signalling will cause immunosuppression and susceptibility to opportunistic infections [11, 12]. The events downstream of CD40 that trigger the PLCγ1–ATP–P2X_7_–proinflammatory cytokine cascade and promotes diabetic retinopathy are unknown. Identification of how CD40 triggers this cascade may enable development of a novel approach to disrupt CD40 without inducing thrombosis or increasing susceptibility to opportunistic infections. This approach could be effective against diabetic retinopathy and numerous inflammatory disorders driven by CD40.

TNF receptor-associated factors (TRAFs) are key mediators of CD40 signalling [13]. CD40 has domains that directly bind TRAF2 and TRAF3 [14] and a site that binds TRAF6 [14]. TRAF3 competes with TRAF2 for binding to CD40, inhibiting CD40 signalling [15]. Expression of CD40 mutants proven not to recruit TRAF2,3 (CD40 ΔT2,3) or TRAF6 (CD40 ΔT6) [16, 17] in human and mouse non-haematopoietic cells, including Müller cells, inhibits CD40-dependent upregulation of ICAM-1 and CCL2 [18, 19]. However, the fact that CD40–TRAF6 drives cellular responses key for protection against infections [19–25] supports the notion that disruption of CD40–TRAF2,3 signalling may inhibit inflammation while preserving cell-mediated immunity, an important consideration since opportunistic infections are complications in individuals with defective CD40–CD154 pathway [26]. We examined the role of TRAF signalling (primarily TRAF2,3 signalling) in activation of the CD40–PLCγ1–ATP–P2X_7_–proinflammatory cytokine cascade and development of diabetic retinopathy. The studies consisted of a combined approach including human Müller cells expressing wild-type (WT) CD40 or CD40 mutants defective in TRAF signalling, transgenic mice expressing in Müller cells either WT CD40 or CD40 mutants and pharmacological disruption of CD40–TRAF2,3 in the diabetic retina.

## Methods

### Transgenic mice

Studies adhered to the institutional guidelines for humane treatment of animals, ‘Principles of laboratory animal care’ (NIH) and to the ARVO Statement for the Use of Animals in Ophthalmic and Vision Research. Studies were approved by the Institutional Animal Care and Use Committee of Case Western Reserve University (IACUC no. 2013-0085). Transgenic mice with conditional expression of WT mouse CD40 restricted to Müller cells have been described [5]. A similar approach was used to generate mice with expression of CD40 mutants (see electronic supplementary material [ESM] Methods for further details). Mutants consisted of mouse CD40 with a mutation that deletes the CD40–TRAF2,3 binding sites [24] and ablates binding to TRAF2 and TRAF3 [16] (CD40 ΔTRAF2,3) and mouse CD40 with previously described point mutations in the TRAF6 binding site (QD*G*Q*A*MED) [24] that prevent binding to TRAF6 [17] (CD40 ΔTRAF6). Heterozygous transgenic mice encoding WT or mutant *Cd40* downstream of Tet^OS^ promoter were bred with homozygous *GFAP*-tTA transgenic mice [27] (all B6/*Cd40*^−/−^ background). Single-transgenic mice served as controls (Trg-Ctr) for double-transgenic mice (Trg-CD40) exhibiting promoter-specific expression of WT or mutant CD40. B6 (000664) and *Cd40*^−/−^ (B6 background; 002928) mice were purchased from The Jackson Laboratory (Bar Harbor, ME, USA). Mice were maintained under identical conditions (12 h light–dark cycle) and were assigned randomly to experimental groups. Each mouse was an experimental unit and samples were assessed blindly.

### Induction of diabetes

Male mice were rendered diabetic by administration of streptozotocin (STZ) [4, 5]. Fasted male mice (20–25 g) received five daily i.p. injections of STZ (55 mg/kg; MP Biomedicals, Solon, OH, USA). Development of diabetes (blood glucose >13.9 mmol/l) was assessed starting 1 week after the first injection of STZ using a Glucometer (Accu-Chek Aviva; Roche Diagnostics, Indianapolis, IN, USA). HbA_1c_ was measured at 2 months (VARIANT Classic; Bio-Rad, Hercules, CA, USA). Mice were weighed weekly and received insulin (0–0.2 units of NPH insulin s.c., 0–3 times per week) if needed. Insulin requirement was similar in all groups.

### Cell-penetrating CD40–TRAF2,3-disrupting peptide

The peptide consisted of the TRAF2,3 binding site of CD40 that was made cell permeable by linkage to TAT_47-57_ cell-penetrating peptide [28]. The peptide was synthesised using d-amino acids following reverse amino acid sequence (retro-inverso [ri] format). The sequence for the CD40–TRAF2,3-disrupting peptide was NH_2_-rrrqrrkkrgy ghlteqv*h*aatn-OH (TAT_47-57_ sequence is underlined). The scrambled peptide NH_2_-rrrqrrkkrgy ntqalahtgevh-OH was used as control. Peptides were manufactured by Biopeptide Co. (San Diego, CA, USA), were low in endotoxin and >98% pure (HPLC). Diabetic B6 mice (2 months) received randomly a single intravitreal injection of the peptides (1 μg).

### Real-time quantitative PCR

Real-time quantitative PCR (qRT-PCR) was performed using primers for *Icam-1* [4], *Ccl2* [18], *Tnf-α* [29], *Il-1β* [30], *Nos2* [31], *P2x*_*7*_ [32] and 18S rRNA [4]. Gene expression was assessed using a 7300 Real-Time PCR System (Applied Biosystems, Waltham, MA, USA). Each cDNA sample was run in triplicate. Samples were normalised according to the content of 18S rRNA.

### Leucostasis

Anesthetised mice were perfused with saline (154 mmol/l NaCl) followed by infusion of 10 ml of PBS containing 200 μl of fluorescein-coupled concanavalin A lectin (5 mg/ml; Vector Laboratories, Burlingame, CA, USA) [9, 33]. Retinal flat-mounts were generated and brightly fluorescent leucocytes adherent to blood vessels were counted in the entire retina using fluorescence microscopy.

### Vascular histopathology

Retinas were dissected from formalin-fixed eyes. After enzyme digestion of the retina [33], neuroretinal tissue was removed and the retinal vasculature was stained with periodic acid-Schiff haematoxylin [33]. Eight areas in the mid-retina were examined blindly using a ZEISS Axio Scan.Z1 (Oberkochen, Germany). Degenerate capillaries were defined as capillary-sized structures without nuclei along their length.

### Immunohistochemistry

Zinc-fixed, paraffin-embedded eyes were treated with proteinase K or citrate buffer. Sections were incubated with primary antibodies listed in ESM Methods. Fluorescent secondary Abs were from Jackson ImmunoResearch Laboratories (West Grove, PA, USA). Antibodies were used at manufacturer-recommended dilutions. Retinas were analysed blindly using an Olympus FV1200 IX-83 confocal microscope (Tokyo, Japan). Images were processed in Photoshop CC 21.0.1 (https://www.adobe.com/products/photoshop.html; accessed 1 April 2021) using similar linear adjustments for all samples. Four or five mice per group were examined (three sections per mouse).

### Cells

The human Müller cell line MIO M1 (gift from Gloria Limb; University College London, UK; identity authenticated by being >95% vimentin-positive, cellular retinaldehyde-binding protein [CRALBP]-positive and glial fibrillary acidic protein [GFAP]-negative) was transduced with previously described retroviral vectors [18, 19] that encode human CD40, hCD40Δ22 (mutant that ablates binding to TRAF2 and TRAF3; hCD40 ΔTRAF2,3) or hCD40EEAA (mutant that prevents binding to TRAF6; hCD40 ΔTRAF6) [16, 17]. Müller cells were also transfected with siRNA against *PLCγ1*, *Src*, *TRAF2*, *TRAF6* or control siRNA. Müller cells were mycoplasma-free and were incubated with multimeric human CD154 to induce CD40 stimulation (1:10 dilution; obtained from Richard Kornbluth, Multimeric Biotherapeutics, La Jolla, CA, USA) or with a non-functional CD154 mutant (T147N) as control [34].

### Immunoblotting

Membranes were probed with primary antibodies listed in ESM Methods followed by incubation with secondary antibody conjugated to horseradish peroxidase (Santa Cruz Biotechnologies; Santa Cruz, CA, USA). Intensities of phosphorylated proteins were calculated using ImageJ (https://imajej.nih.gov; accessed 2 February 2019) and normalised against respective total proteins.

### RNAi-mediated silencing

Transfection with siRNA was used for gene knockdown. See ESM Methods.

### Flow cytometry

Expression of surface molecules in Müller cells was assessed by flow cytometry. See ESM Methods for details.

### Measurement of extracellular

ATP was quantified using a bioluminescence assay. See ESM Methods for details.

### Statistical analysis

Results were expressed as the mean ± SEM or mean ± SD as indicated. Statistical significance of in vitro experiments were analysed by two-tailed Student’s *t* test or ANOVA. In vivo experiments were analysed using the non-parametric Kruskal–Wallis test followed by the Mann–Whitney test. Differences were considered statistically significant at *p*<0.05.

## Results

### Effect of mutations in CD40–TRAF binding sites on PLCγ1 activation and ATP release in Müller cells and the release of proinflammatory cytokines in bystander myeloid cells

**Fig. 1:**
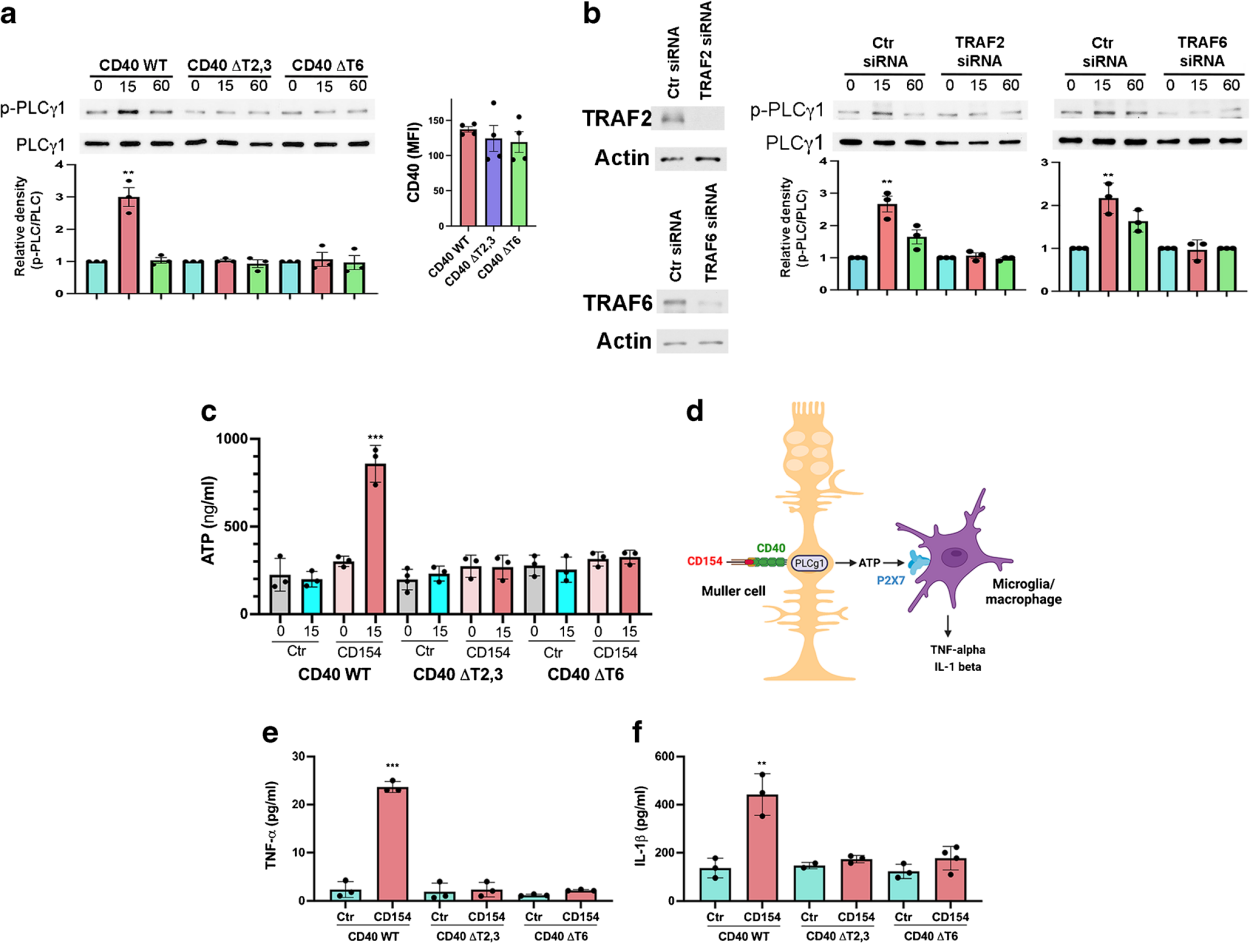
Effect of disruption of CD40–TRAF signalling on PLCγ1 activation and ATP release in Müller cells as well as release of TNF-α and IL-1β in bystander myeloid cells. (**a**) Human Müller cells transduced with retroviral vector encoding WT CD40, CD40 ΔT2,3 or CD40 ΔT6. CD40 expression was examined by FACS. Cells were incubated with CD154 for 15 or 60 min. Phospho-Tyr783 PLCγ1 and total PLCγ1 were assessed by immunoblot. Relative density of phospho-Tyr783 PLCγ1 for each cell type was normalised to total PLCγ1 followed by normalisation relative to their respective unstimulated control samples (0 min time point). Relative density of phospho-Tyr783 PLCγ1 for unstimulated samples was given a value of 1. Graphs represent quantification of phospho-Tyr783 PLCγ1 relative to total PLCγ1 from three different experiments. (**b**) Müller cells expressing WT CD40 were transfected with siRNA against *TRAF2* or *TRAF6* or with control siRNA. Expression of TRAF2, TRAF6 and actin were assessed by immunoblot. Müller cells were stimulated with CD154 for 15 and 60 min. Phospho-Tyr783 PLCγ1 and total PLCγ1 were assessed as in (**a**). Relative densities of phospho-Tyr783 PLCγ1 from cells transfected with control, *TRAF2* or *TRAF6* siRNA were compared with bands from their respective unstimulated cells. Graphs represent quantification from three different experiments. (**c**) Supernatant fractions were collected at 0 and 15 min after incubation with CD154 and used to measure extracellular ATP (*n*=3). (**d**) CD40 stimulation in Müller cells triggers proinflammatory cytokine production by myeloid cells. CD40 ligation in Müller cells activates PLCγ1 that in turn triggers secretion of extracellular ATP. The P2X_7_ receptor is upregulated in microglia/macrophages in the diabetic retina. ATP binds P2X_7_ receptor leading to secretion of TNF-α and IL-1β. Created with BioRender.com (with permission). (**e**, **f**) Müller cells were incubated with CD40^−^ human monocytic cell lines (MonoMac6) with or without CD154. TNF-α (**e**) and IL-1β (**f**) were measured by ELISA at predetermined optimal time points (4 h for TNF-α and 24 h for IL-1β). Results are presented as mean ± SD (*n*=3). **p<0.01 and ***p<0.001 by Student’s *t* test. Ctr, control; MFI, mean fluorescence intensity

PLCγ1 mediates ATP release induced by CD40 in Müller cells [5]. In contrast, PLCγ1 knockdown did not inhibit upregulation of proinflammatory molecules CCL2 and ICAM-1 induced by CD40 ligation in Müller cells (ESM Fig. 1a). Moreover, PLCγ1 knockdown did not impair CD40-induced activation of extracellular signal-regulated kinase (ERK), c-Jun N-terminal kinase (JNK), p38 mitogen-activated protein kinase (MAPK) and NF-κB, molecules that cooperate to upregulate CCL2 and ICAM-1 [35, 36] (ESM Fig. 1b). Although the studies suggest that proinflammatory molecule upregulation and the release of extracellular ATP induced by CD40 in Müller cells are controlled by pathways that are at least partially non-overlapping, it was still possible that both responses are controlled by CD40–TRAF interaction, the upstream event in CD40 signalling. We tested whether the CD40–TRAF pathways are required for PLCγ1 activation and the release of extracellular ATP. Stimulation with CD154 activated PLCγ1 in human Müller cells that express WT CD40 (Fig. 1a). This response was impaired in cells that express CD40 mutants that do not recruit TRAF2,3 (CD40 ΔT2,3) or TRAF6 (CD40 ΔT6) (Fig. 1a). Given the possibility that disruption of the cytoplasmic domain of CD40 may impair interactions with non-TRAF proteins, we confirmed the results using TRAF2- and TRAF6-deficient cells (Fig. 1b). Disruption of CD40–TRAF2,3 or CD40–TRAF6 signalling also impaired release of extracellular ATP (Fig. 1c). These findings, together with the evidence that CD40–TRAF signalling is required for CCL2/ICAM-1 upregulation in Müller cells [18], support the notion that CD40–TRAF interaction regulates PLCγ1 activation, ATP release and proinflammatory molecule upregulation in these cells.

Müller cells do not secrete TNF-α and IL-1β in response to CD154 [5]. However, CD154 triggers release of extracellular ATP that causes P2X_7_-dependent production of proinflammatory cytokines in myeloid cells [5] (Fig. 1d). We examined the effects of CD40–TRAF signalling in Müller cells on proinflammatory cytokine production by myeloid cells. Human Müller cells that express WT CD40, CD40 ΔT2,3 or CD40 ΔT6 were incubated with MonoMac6 cells. These *CD40*^−/−^ monocytic cells secrete proinflammatory cytokines in response to ATP but not CD154 [5]. CD40 stimulation of human Müller cells expressing WT CD40 triggered secretion of TNF-α and IL-1β by MonoMac6 cells (Fig. 1e,f), a response known to be driven by ATP release by Müller cells [5]. Proinflammatory cytokine secretion was impaired when CD40 ΔT2,3- or CD40 ΔT6-expressing Müller cells were incubated with MonoMac6 (Fig. 1e,f). Thus, CD40 with mutations in the TRAF2,3 or TRAF6 binding sites fail to activate the CD40–PLCγ1–ATP–TNF-α/IL-1β secretion cascade in cells deficient in WT CD40.

### Role of Src in CD40–TRAF-dependent activation of PLCγ1

**Fig. 2:**
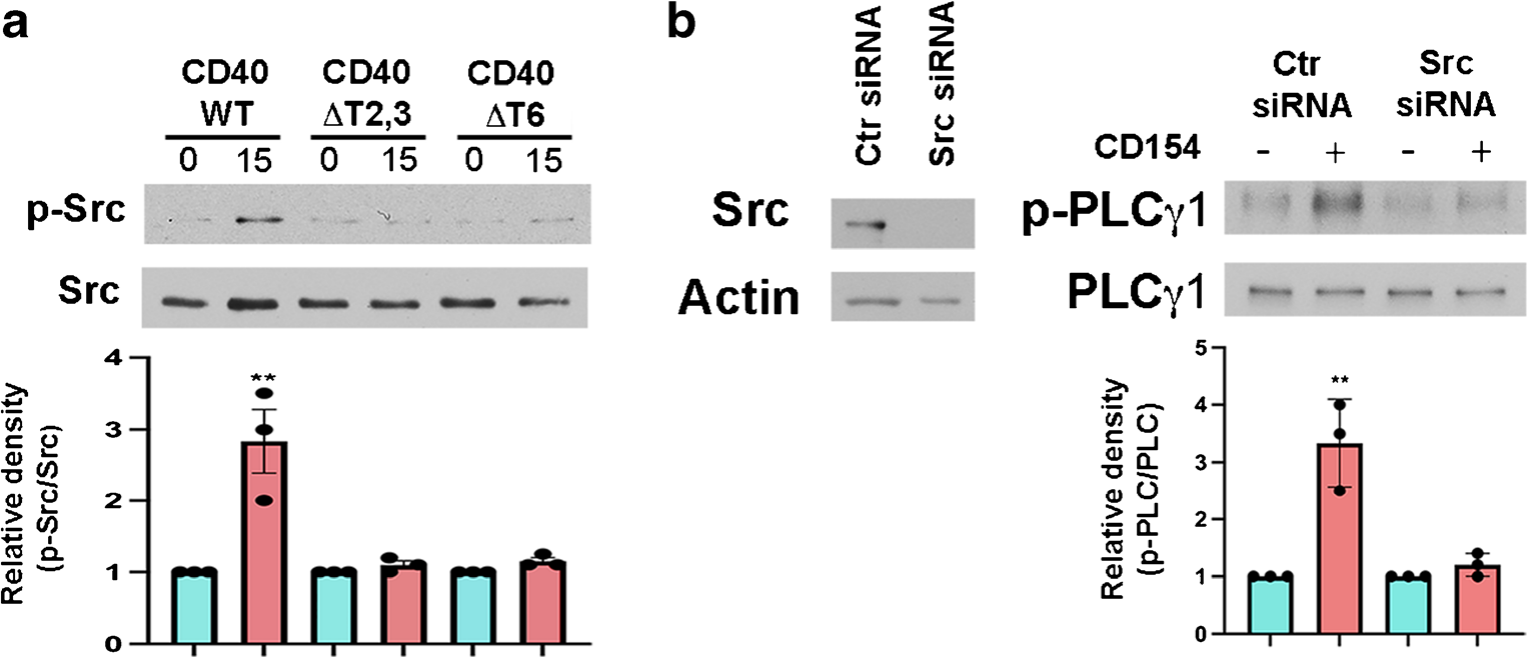
Role of Src as a molecular link between CD40–TRAF signalling and activation of PLCγ1. (**a**) Human Müller cells that express WT CD40, CD40 ΔT2,3 or CD40 ΔT6 were incubated with CD154 for 15 min. Phospho-Tyr416 Src and total Src were assessed by immunoblot. Relative density of phospho-Tyr416 Src for each cell type was determined as described in Fig. 1. Graphs represent quantification of phospho-Tyr416 Src relative to total Src from three different experiments. (**b**) Müller cells that express WT CD40 were transfected with siRNA against *Src* or with control siRNA. Expression of Src and actin were assessed by immunoblot. Müller cells were incubated with CD154. Phospho-Tyr783 PLCγ1 and total PLCγ1 were examined by immunoblot at 15 min. Graphs represent quantification of phospho-Tyr783 PLCγ1 relative to total PLCγ1 from three different experiments. Results are presented as mean ± SD (*n*=3). **p<0.01 by Student’s *t* test. Ctr, control

We examined whether Src links CD40–TRAFs to PLCγ1 signalling since Src can activate PLCγ1 and CD40 induces Src signalling [21, 37]. CD40 ligation induced Src activation in Müller cells that express WT CD40 but not in cells that express CD40 ΔT2,3 or CD40 ΔT6 (Fig. 2a). Moreover, knockdown of Src in WT CD40-expressing Müller cells impaired CD40-dependent PLCγ1 activation (Fig. 2b). Thus, CD40–TRAF signalling activates PLCγ1 in an Src-dependent manner.

### Effect of CD40 ΔT2,3 expressed in Müller cells on CCL2 upregulation in the diabetic retina

**Fig. 3:**
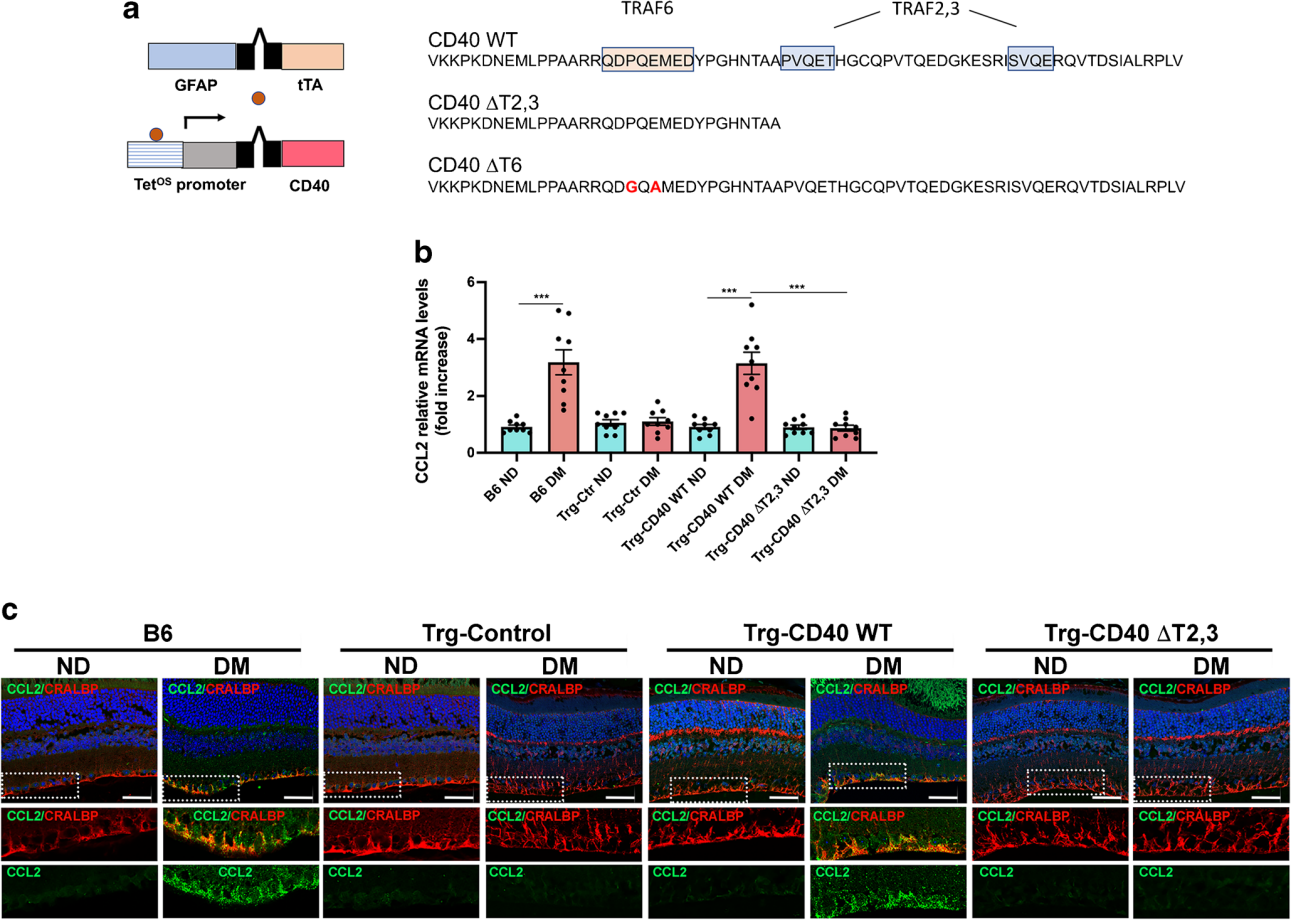
Effect of CD40 ΔT2,3 expressed in Müller cells from diabetic mice on upregulation of CCL2, an inflammatory molecule that is normally directly induced by WT CD40 in Müller cells. (**a**) Lines of transgenic mice. Responder lines of mice have a transgene for either WT *Cd40*, *Cd40* ΔT2,3 or *Cd40* ΔT6 cloned downstream of the Tet^OS^ promoter. Driver mice have a tTA transgene downstream of *GFAP* promoter. Amino acid sequences of the corresponding intracytoplasmic tail of CD40 are shown. Amino acids in red represent mutations known to impair recruitment of TRAF6. (**b**) At 2 months of diabetes, retinas from diabetic B6, Trg-Ctr, Trg-CD40 WT and Trg-CD40 ΔT2,3 mice, as well as from non-diabetic control mice, were collected and used for mRNA extraction. *Ccl2* mRNA was assessed by real-time quantitative PCR using 18S rRNA as internal control. One non-diabetic B6 mouse was given an arbitrary value of 1 and data are expressed as fold increase compared with this mouse. Bars represent mean ± SEM (*n*=7–9 mice per group). ***p<0.01 by ANOVA. (**c**) Retinal sections were incubated with antibodies against CCL2 and CRALBP (which labels Müller cells). Areas within the boxes are magnified in lower images. Scale bar, 50 μm. DM, diabetic; ND, non-diabetic

Transgenic mice were generated by crossing the following mice: (1) Driver *Cd40*^−/−^ mice homozygous for the tTA transgene under the control of a promoter that drives gene expression in Müller cells (human *GFAP* promoter gfa2; *GFAP*-tTA mice); and (2) Responder *Cd40*^−/−^ mice containing transgenes of WT *Cd40*, *Cd40* ΔT2,3 or *Cd40* ΔT6, all of them cloned downstream of a Tet^OS^ promoter (Fig. 3a). While single-transgenic mice (carrying only the *GFAP*-tTA) do not exhibit rescue of CD40, double-transgenic mice have been demonstrated to express CD40 in Müller cells but not in astrocytes, endothelial cells, microglia, ganglion cells or leucocytes [5]. Immunohistochemistry confirmed CD40 expression in CRALBP-positive cells (Müller cells) from double-transgenic Trg-CD40 WT, Trg-CD40 ΔT2,3 and Trg-CD40 ΔT6 mice but not in single-transgenic (Trg-Ctr) mice (ESM Fig. 2a). Moreover, flow cytometry analysis of CD29^+^ cells (Müller cells) revealed that CD40 expression was similar among B6, Trg-CD40 WT, Trg-CD40 ΔT2,3 and Trg-CD40 ΔT6 mice (ESM Fig. 2b, c).

We focused on the role of CD40–TRAF2,3 for the following reasons: (1) disruption of this pathway markedly inhibits the CD40–PLCγ1–ATP–TNF-α/IL-1β cascade and proinflammatory responses directly induced by CD40 in Müller cells [18]; and (2) while the CD40–TRAF6 pathway also promotes inflammation, disruption of this pathway impairs mechanisms that control opportunistic pathogens [19]. Indeed, disruption of the CD40–TRAF6 (but not CD40–TRAF2,3) pathway increases susceptibility to retinitis caused by *Toxoplasma gondii*, a pathogen that chronically infects one-third of the world population and is a major cause of infectious retinitis worldwide [28].

We examined the in vivo effects of disruption of CD40–TRAF2,3 signalling on the expression of CCL2, a molecule that CD40 directly upregulates in Müller cells [18]. Male B6, Trg-Ctr, Trg-CD40 WT and Trg-CD40 ΔT2,3 mice were made diabetic using STZ. Blood glucose concentrations, HbA_1c_ levels and body weights were similar among all groups of diabetic mice (ESM Table 1) (*p*>0.2). Diabetic Trg-CD40 WT mice displayed upregulated *Ccl2* mRNA levels (Fig. 3b). This was accompanied by increased expression of CCL2 in Müller cells (Fig. 3c). In contrast, diabetic Trg-CD40 ΔT2,3 mice did not display increased *Ccl2* retinal mRNA levels and did not increase CCL2 protein expression in Müller cells (Fig. 3b,c). Thus, in vivo expression of the CD40 ΔT2,3 mutant fails to support upregulation of an inflammatory molecule in Müller cells from diabetic mice deficient in WT CD40.

### Effect of CD40 ΔT2,3 on PLCγ1 activation in Müller cells in the diabetic retina

**Fig. 4:**
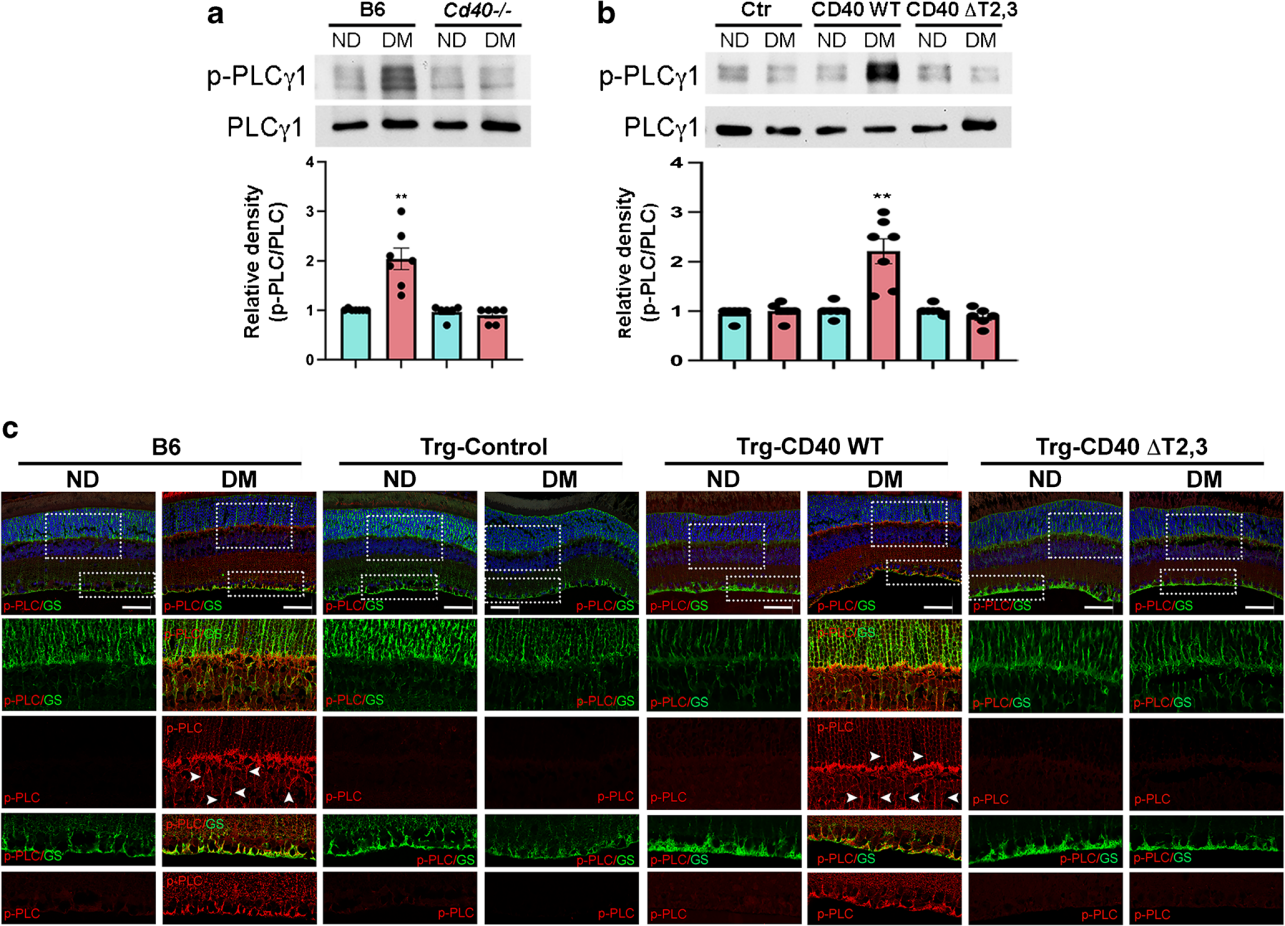
Effect of CD40 ΔT2,3 expressed in Müller cells from diabetic mice on Tyr783 phosphorylation of PLCγ1. (**a**, **b**) At 2 months of diabetes, retinal lysates from B6 and *Cd40*^−/−^ mice (**a**) or transgenic Trg-CD40 WT and Trg-CD40 ΔT2,3 mice (**b**) were probed for expression of phospho-Tyr783 PLCγ1 and total PLCγ1 by immunoblot. Graphs represent quantification of phospho-Tyr783 PLCγ1 relative to total PLCγ1 from 4–7 mice per group. (**c**) Retinal sections were incubated with antibodies against phospho-Tyr783 PLCγ1 and glutamine synthetase (which labels Müller cells). Areas within the boxes are magnified in lower images. Arrowheads show some of the areas where phospho-Tyr783 PLCγ1 co-localises with glutamine synthetase. Scale bar, 50 μm. **p<0.01 by Student’s *t* test. DM, diabetic; GS, glutamine synthetase; ND, non-diabetic

We examined the effects of diabetes and the CD40–TRAF2,3 pathway on PLCγ1 activation. Retinal lysates of diabetic B6 mice revealed increased phosphorylation of PLCγ1 compared with non-diabetic control mice (Fig. 4a). This response was not observed in *Cd40*^*−/−*^ mice (Fig. 4a). Rescue of WT CD40 in Müller cells (Trg-CD40 WT mice) restored phosphorylation of PLCγ1 in retinal lysates of diabetic mice (Fig. 4b). This was accompanied by increased PLCγ1 phosphorylation in Müller cells (Fig. 4c). PLCγ1 activation was disrupted in diabetic Trg-CD40 ΔT2,3 mice (Fig. 4b,c). Thus, expression of the CD40 ΔT2,3 mutant does not support PLCγ1 activation in Müller cells from diabetic mice deficient in WT CD40.

### Effect of CD40 ΔT2,3 on upregulation of *P2x*_*7*_, *Tnf-α*, *Il-1β* and *Nos2* mRNA levels in the diabetic retina

**Fig. 5:**
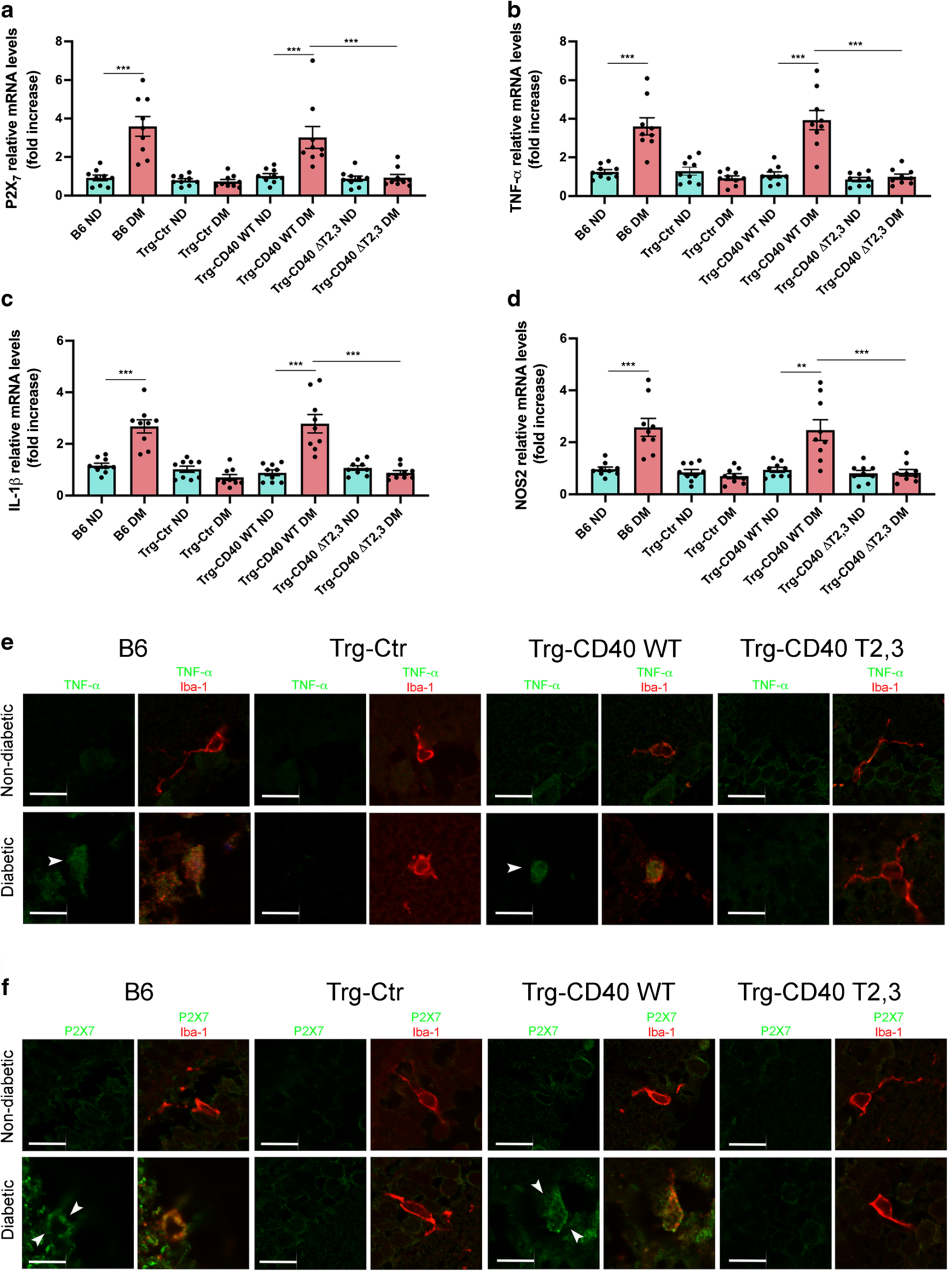
Effect of CD40 ΔT2,3 expressed in Müller cells from diabetic mice on upregulation of P2X_7_, TNF-α, IL-1β, and NOS2 in the retina. (**a**–**d**) At 2 months of diabetes, inflammatory molecules’ mRNAs were assessed by real-time quantitative PCR using 18S rRNA as internal control. One non-diabetic B6 mouse was given an arbitrary value of 1 and data are expressed as fold increase compared with this mouse. Bars represent mean ± SEM (*n*=7–9 mice per group). **p<0.01 and ***p<0.001 by ANOVA. (**e**, **f**) Retinal sections were incubated with anti-TNF-α plus anti-Iba-1 (a marker of microglia/macrophages) (**e**) or anti-P2X_7_ plus anti-Iba-1 (**f**). Arrowheads show TNF-α-positive or P2X_7_-positive areas that co-localise with Iba-1. Scale bar, 10 μm. DM, diabetic; ND, non-diabetic

CD40 expressed in Müller cells in the diabetic retina induces inflammatory molecule expression in myeloid cells through P2X_7_ [5]. Moreover, in the presence of diabetes, B6 mice and Trg-CD40 WT mice upregulate *P2x*_*7*_ mRNA levels [5], consistent with the notion that P2X_7_ upregulation accompanies and facilitates in vivo purinergic signalling. In contrast, diabetic Trg-CD40 ΔT2,3 mice did not display upregulated *P2x*_*7*_ mRNA levels (Fig. 5a). Next, we examined the effects of CD40 ΔT2,3 on expression of TNF-α, IL-1β and NOS2, inflammatory molecules driven by the P2X_7_ receptor in the diabetic retina [5]. While diabetic Trg-CD40 WT mice displayed upregulated mRNA of these inflammatory molecules, no upregulation was noted in diabetic Trg-CD40 ΔT2,3 mice (Fig. 5b–d). Moreover, microglia/macrophages from these mice did not exhibit increased expression of P2X_7_ and TNF-α (Fig. 5e,f). Altogether, expression of CD40 ΔT2,3 in Müller cells fails to support P2X_7_ receptor, TNF-α, IL-1β and NOS2 upregulation in the retina of diabetic mice deficient in WT CD40.

While we focused on the relevance of CD40–TRAF2,3 signalling, we also began to explore the role of the CD40–TRAF6 pathway in the development of inflammatory responses. In contrast to Trg-CD40 WT mice, diabetic Trg-CD40 ΔT6 mice did not display upregulation of *P2x*_*7*_, *Tnf-α*, *Il-1β*, *Nos2*, *Icam-1* or *Ccl2* mRNA (ESM Fig. 3). Thus, expression of CD40 ΔT6 in Müller cells does not promote upregulation of proinflammatory molecules in the retina of diabetic mice deficient in WT CD40.

### Effect of CD40 ΔT2,3 on ICAM-1 upregulation, leucostasis and development of diabetic retinopathy

**Fig. 6:**
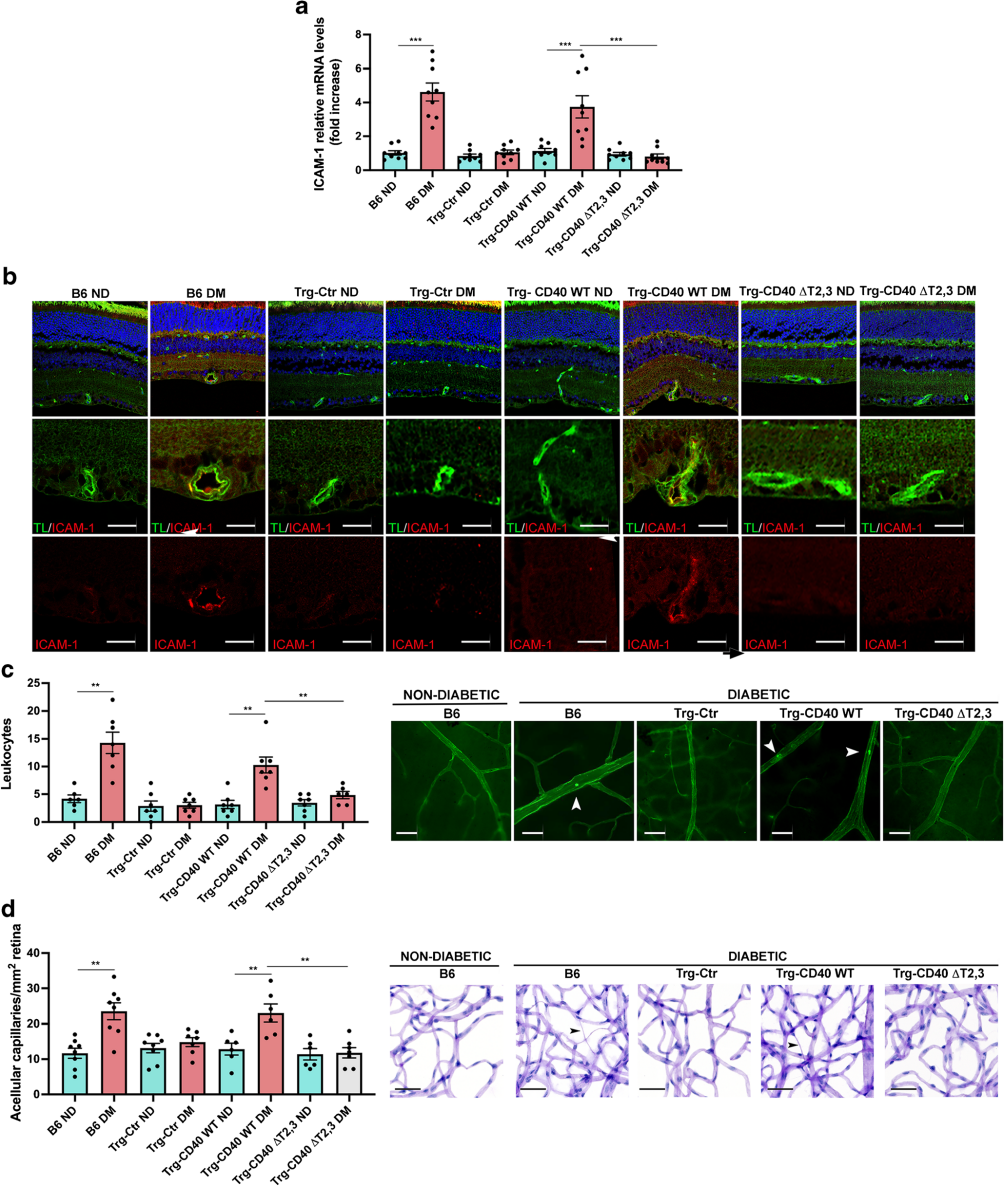
Effect of CD40 ΔT2,3 expressed in Müller cells from diabetic mice on upregulation of ICAM-1 in the retina and development of early diabetic retinopathy. (**a**) At 2 months of diabetes, *Icam-1* mRNA was assessed by real-time quantitative PCR in retinas from diabetic B6, Trg-Ctr, Trg-CD40 WT and Trg-CD40 ΔT2,3 mice as well as from non-diabetic control mice. 18S rRNA was used as internal control. One non-diabetic B6 mouse was given an arbitrary value of 1 and data are expressed as fold increase compared with this mouse. Bars represent mean ± SEM (*n*=7–9 mice per group). (**b**) At 2 months of diabetes, retinal sections were incubated with anti-ICAM-1 mAb plus tomato lectin (labels neural mouse endothelial cells). Magnified blood vessels are shown. Scale bar, 10 μm. (**c**) At 2 months of diabetes, concanavalin A-labelled adherent leucocytes in the retinal vasculature were quantified. Retinal flat-mounts were generated and brightly fluorescent leucocytes adherent to blood vessels were counted in the entire retina using fluorescence microscopy. Arrowheads show adherent leucocytes within the vasculature. Scale bar, 20 μm. *n*=6 or 7 mice per group. (**d**) At 8 months of diabetes retinal digests were examined for the presence of degenerate capillaries. Bars represent mean ± SEM. *n*=6–8 mice per group. Arrows show degenerate capillaries in the retinal digest. Scale bar, 50 μm. **p<0.01 and ***p<0.001 by ANOVA. DM, diabetic; ND, non-diabetic; TL, tomato lectin

ICAM-1 upregulation in retinal endothelial cells and leucostasis are important features of diabetic retinopathy. Diabetic Trg-CD40 WT mice displayed upregulated *Icam-1* mRNA similarly to diabetic B6 mice (Fig. 6a). This was accompanied by increased expression of ICAM-1 in retinal capillaries (Fig. 6b). In contrast, expression of CD40 ΔT2,3 prevented upregulation of *Icam-1* mRNA and ICAM-1 protein (Fig. 6a,b). Moreover, an increase in the numbers of adherent leucocytes (leukostasis) was noted in diabetic Trg-CD40 WT mice but not in diabetic Trg-CD40 ΔT2,3 mice (Fig. 6c).

The transformation of retinal capillaries into basement membrane tubes that lack cells and blood flow (degenerate capillaries) is a central feature of early diabetic retinopathy. Compared with diabetic B6 and Trg-CD40 WT mice, diabetic Trg-CD40 ΔT2,3 mice did not develop capillary degeneration (Fig. 6d). Altogether, replacing WT CD40 with CD40 ΔT2,3 in Müller cells is sufficient to disrupt vascular inflammatory responses in the diabetic retina and the development of experimental diabetic retinopathy.

### Effect of pharmacological inhibition of CD40–TRAF2,3 signalling on upregulation of P2X_7_, inflammatory molecules and leucostasis in the diabetic retina

**Fig. 7:**
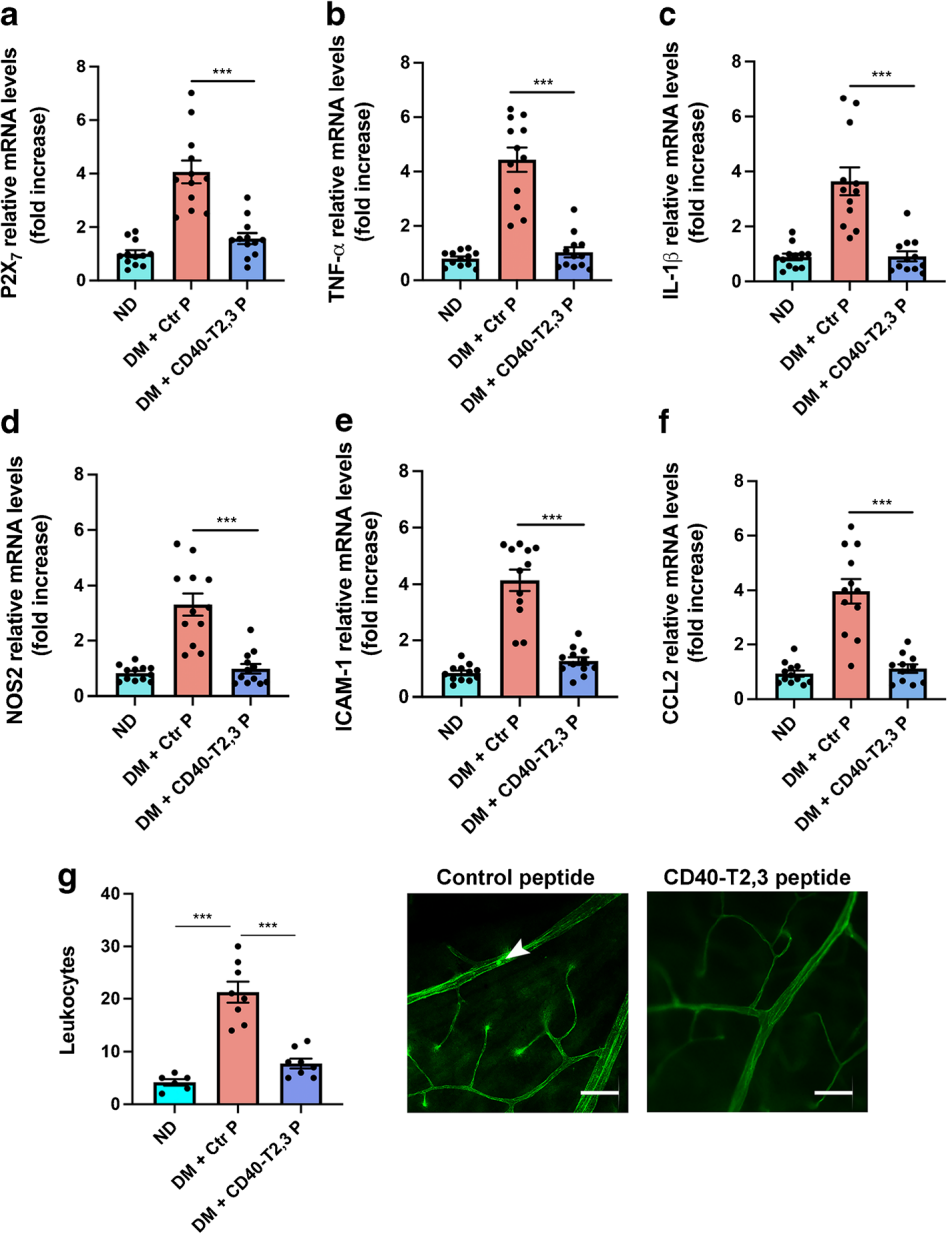
Effect of administration of a CD40–TRAF2,3 peptide on upregulation of P2X_7_ and inflammatory molecules as well as leucostasis in the diabetic retina. At 2 months of diabetes, one eye from each B6 mouse received either ri control peptide or ri CD40–TRAF2,3 peptide (1 μg, by intravitreal injection). Eyes were collected after 2 weeks. (**a**–**f**) *P2x*_*7*_ (**a**), *Tnf-α* (**b**), *Il-1β* (**c**), *Nos2* (**d**), *Icam-1* (**e**) and *Ccl2* mRNAs (**f**) were assessed by quantitative real-time PCR. (**g**) Adherent leucocytes in the retinal vasculature were quantified by labelling with concanavalin A. Horizontal bars represent mean ± SEM (*n*=6–12 mice per group). Arrowhead shows adherent leucocyte within the vasculature in retinal flat-mount. Scale bar, 20 μm. ***p<0.001 by ANOVA. CD40-T2,3 ri CD40–TRAF2,3 peptide; Ctr P, ri control peptide; DM, diabetic; ND, non-diabetic

We developed a pharmacological approach to inhibit CD40–TRAF2,3 signalling that consisted of a cell-permeable peptide containing the amino acid sequence of the TRAF2,3 binding site of CD40 fused with HIV TAT_47-57_ [28]. The peptide is synthesised with d-amino acids in a reverse amino acid sequence (retro-inverso; ri) to make it resistant to peptidases while maintaining the ability to disrupt CD40–TRAF2,3 signalling [28]. The ri CD40–TRAF2,3 peptide inhibits CD40-driven proinflammatory responses in vitro and translocates into retinal cells, including Müller cells, following intravitreal injection [28]. B6 mice that had been diabetic for 2 months were injected intravitreally with ri CD40–TRAF2,3 or ri control peptide. The ri CD40–TRAF2,3 peptide impaired upregulation of *P2x*_*7*_, *Tnf-α*, *Il-1β*, *Nos2*, *Icam-1* and *Ccl2* mRNA (Fig. 7a–f). Moreover, the ri CD40–T2,3 peptide impaired leucostasis (Fig. 7g). Thus, a pharmacological approach that disrupts the CD40–TRAF2,3 pathway impairs upregulation of P2X_7_ and proinflammatory molecules in the retina as well as retinal leucostasis in diabetic mice.

## Discussion

CD40 expressed in Müller cells is pivotal for the development of diabetic retinopathy [5]. Through ATP release, CD40 signalling in retinal Müller cells triggers in myeloid cells P2X_7_-dependent expression of proinflammatory molecules involved in the pathogenesis of diabetic retinopathy [5]. We uncovered that disruption of CD40–TRAF2,3 signalling inhibited the CD40–PLCγ1–P2X_7_–proinflammatory molecule pathway in the diabetic retina. Src acted as a link between CD40–TRAF2,3 and PLCγ1 activation. Disrupting CD40–TRAF2,3 interaction had the following effects: (1) impairment of CD40-driven PLCγ1 activation in human Müller cells in vitro, inhibiting ATP release and secretion of TNF-α and IL-1β in myeloid cells; (2) inhibition of in vivo expression of activated PLCγ1 in Müller cells and expression of P2X_7_ and TNF-α in microglia/macrophages; (3) impairment of upregulation of *P2x*_*7*_, *Tnf-α*, *Il-1β*, *Nos2*, *Icam-1* and *Ccl2* mRNA in the diabetic retina; (4) and inhibition of upregulation of ICAM-1 in endothelial cells and leucostasis, responses driven by proinflammatory cytokines and NOS2 [38–40]. Importantly, replacing WT CD40 by CD40 ΔT2,3 was sufficient to prevent the development of diabetic retinopathy. Moreover, the studies with a peptide that impedes CD40–TRAF2,3 signalling revealed that targeting this pathway pharmacologically reduced inflammation in the diabetic retina.

P2X_7_ drives ATP-induced cytokine production by macrophages/microglia [41–44]. Moreover, P2X_7_ plays a central role in the pathogenesis of diabetic retinopathy. P2X_7_ is upregulated in the retina and microglia/macrophages of diabetic B6 and Trg-CD40 WT mice [5]. This finding is likely relevant since increased P2X_7_ expression promotes receptor function [45]. In addition, the absence of P2X_7_ (*P2x*_*7*_^−/−^) in diabetic mice or the administration of the P2X_7_ inhibitor Brilliant blue G (BBG) to diabetic mice prevents upregulation of TNF-α, IL-1β, NOS2 and ICAM-1 [5]. Purinergic-dependent cytokine production by microglia/macrophages enables Müller cells to bypass their poor ability to secrete proinflammatory cytokines in response to CD40 ligation [5]. Moreover, this mechanism explains why disruption of CD40–TRAF2,3 signalling is effective in impairing expression of proinflammatory cytokines. While both CD40–TRAF2,3 and CD40–TRAF6 signalling are required for adhesion molecule upregulation and chemokine production induced by CD40 in non-haematopoietic cells, CD40–TRAF2,3 plays a secondary role in CD40-driven production of proinflammatory cytokines in myeloid cells [18, 19]. The discovery that CD40–TRAF2,3 is required to activate ATP release in Müller cells likely explains why targeting this pathway impairs upregulation of the expression of various inflammatory molecules including TNF-α and IL-1β.

Besides inducing ATP release that triggers P2X_7_-dependent expression of proinflammatory molecules in myeloid cells, CD40 signalling in Müller cells also directly upregulates proinflammatory molecules in these cells (e.g. CCL2 and ICAM-1) [18]. Our studies suggest that the signalling pathways downstream of CD40 that control these two types of responses do not overlap, since PLCγ1 is required for ATP release and proinflammatory cytokine production by myeloid cells but is dispensable for CD40-driven CCL2/ICAM-1 upregulation and activation of signalling cascades known to drive CCL2 and ICAM-1 expression (ERK, JNK, p38 MAPK and NF-κB). Despite these differences, disruption of CD40–TRAF2,3 signalling inhibited the pathway of PLCγ1–ATP–P2X7–proinflammatory molecules in myeloid cells and inhibited the expression of inflammatory molecules that CD40 directly upregulates in Müller cells [18]. These findings support the critical role of the CD40–TRAF2,3 pathway as inducer of various inflammatory responses.

Although rodent models of diabetic retinopathy do not allow the examination of macular abnormalities and neovascularisation, animal models advanced our understanding of the pathogenesis of diabetic retinopathy as it relates to humans [2, 3]. Moreover, genetic manipulations in mice that target pathways of interest provide a unique tool for precise examination of their role in diabetic retinopathy. These approaches, together with pharmacological inhibition of these pathways in animals and in vitro studies in human retinal cells, represent a robust initial approach to identify novel targets against the disease.

The CD40–CD154 pathway is a therapeutic target against inflammatory disorders [1]. However, the thrombotic complications of anti-CD154 mAbs and the likely side-effects of generalised inhibition of CD40 signalling indicate that new approaches to impair CD40-induced inflammation are required. Targeting CD40–TRAF interaction is an attractive approach. Prior studies have centred on inhibition of CD40–TRAF6 signalling [46, 47]. Indeed, disruption of CD40–TRAF6 signalling reduced inflammation in the diabetic retina. However, this approach impairs cellular mechanisms of protection against infections, a concern since opportunistic infections frequently afflict individuals with deficiency in CD40–CD154 signalling [26]. Our studies indicate that disrupting the CD40–TRAF2,3 pathway may become a novel approach to inhibit inflammatory responses involved in the pathogenesis of diabetic retinopathy, and potentially other CD40-driven inflammatory diseases. This approach will likely avoid thrombotic events caused by crosslinking of CD154 and avoid impairing cellular immunity induced by global inhibition of CD40 signalling (anti-CD40 mAb, anti-CD154 Fab antibody) or inhibition of CD40–TRAF6 signalling.

## Supplementary information

ESM 1 (PDF 1444 kb)

## Data Availability

All data generated and analysed during this study are included in this published article.

## Abbreviations

CCL2: C-C motif chemokine ligand 2
CRALBP: Cellular retinaldehyde-binding protein
ERK: Extracellular signal-regulated kinase
GFAP: Glial fibrillary acidic protein
ICAM-1: Intercellular adhesion molecule 1
JNK: c-Jun N-terminal kinase
mAb: Monoclonal antibody
MAPK: Mitogen-activated protein kinase
NOS2: Nitric oxide synthase 2
PLCγ1: Phospholipase Cγ1
ri: Retro-inverso
STZ: Streptozotocin
TRAF: TNF receptor-associated factor
WT: Wild-type
