# Supplementary material for: Disruption of retinal inflammation and the development of diabetic retinopathy in mice by a CD40-derived peptide or mutation of CD40 in Müller cells
Source: Diabetologia. 2022 Aug 3;65(12):2157–71. doi: 10.1007/s00125-022-05775-6 (PMC9630214; doi:10.1007/s00125-022-05775-6)
Supplement: Supplementary file 1 — (PDF 1444 kb) [file 125_2022_5775_MOESM1_ESM.pdf]

Disruption of retinal inflammation and the development of diabetic retinopathy in mice by a CD40-derived peptide or mutation of CD40 in Müller cells

Jose-Andres C. Portillo[1], Jin-Sang Yu[1], Sarah Vos[1], Reena Bapputty[2], Yalitza Lopez Corcino[1], Alyssa Hubal[1,3], Jad Daw[1], Sahil Arora[1], Wenyu Sun[4], Zheng-Rong Lu[4] and Carlos S. Subauste[1,3]

[1]Division of Infectious Diseases and HIV Medicine, Department of Medicine, Case Western Reserve University, Cleveland, OH, USA

[2]Department of Pediatrics, Case Western Reserve University, Cleveland, OH, USA

[3]Department of Pathology, Case Western Reserve University, Cleveland, OH, USA

[4]Department of Biomedical Engineering, Case Western Reserve University, Cleveland, OH, USA

ESM METHODS

Transgenic mice

Transgenic mice with conditional expression of WT mouse CD40 restricted to Müller cells were previously described [1]. A similar approach was used to generate transgenic mice with conditional expression of CD40 mutants. Briefly, constructs that encode mouse WT *Cd40*, mouse *Cd40* with a previously described mutation that deletes the CD40-TRAF2,3 binding sites [2] and ablates binding to TRAF2 and TRAF3 [3] (CD40 ΔTRAF2,3), or mouse CD40 with previously described point mutations in the TRAF6 binding site (QD*G*Q*A*MED) [2] that prevents binding to TRAF6 [4] (CD40 ΔTRAF6) were inserted into the *Eco* RI and *Bam* HI sites of the pTet^{os} plasmid [5]. After sequence verification, transgenes were excised by *Sal* I digestion [5]. Transgenes were microinjected into mouse oocytes (C57BL/6J; B6). Founder Tet^{OS}-*Cd40* mice (either WT or mutant *Cd40*) were identified by PCR using the primers: Tet^{OS}*Cd40* Forward: 5'-GCAACGTGCTGGTTATTGTG-3', Reverse: 5'-CCGGGACTTTAAACCACAGA-3'. The driver line consisted of transgenic mice that express tetracycline (Tet)-repressible transactivator (tTA) under the control of the *GFAP* promoter *gfa2* [6]. Homozygous Tet^{OS}-*Cd40* encoding either WT or mutant *Cd40* (responder) and heterozygous *GFAP*-tTA (driver) transgenic mice [6] (both B6) were backcrossed onto a *Cd40*$^{-/-}$ (B6) background. To confirm that mice were *Cd40*$^{-/-}$, animals were genotyped using primers that detect endogenous *Cd40* and mutant *Cd40* (neomycin cassette inserted into exon 3 resulting in lack of functional Cd40) [7]. Both lines of mice were bred and offspring identified by PCR analysis of genomic DNA. PCR primers for *Cd40* and tTA were obtained from The Jackson Laboratory (Bar Harbor, ME). Littermates that inherited only one transgene (single transgenic and non-expressing) served as controls (Trg-Ctr) for double transgenic animals (Trg-CD40; exhibiting promoter-specific expression of WT or mutant CD40).

Antibodies

Retinal sections were incubated with biotinylated anti-CD40 mAb (3/23, BioLegend, San Diego, CA; 1:50), anti-CRALBP (15356-1-AP, Proteintech Group, Rosemont, IL; 1:200), anti-vimentin (Novus Biologicals, Littleton, CO; 1:300), anti-glutamine synthetase (GTX630654, GeneTex, Irvine, CA; 1:1000), anti-Iba-1 Ab (019-19741, Wako Chemicals, Richmond, VA; 1:250), Tomato Lectin DyLight 488 (Vector Laboratories, Burlingame, CA; 1:100), anti-ICAM-1 (KAT-1, eBiosciences, San Diego, CA; 1:100), anti-TNF-α (52B83, Abcam, Cambridge, UK; 1:25), anti-CCL2 Ab (2D8, Novus Biologicals; 1:1000), anti-$P2X_7$ (AP09521PU-N, Origene, Rockville, MD; 1:100) or biotinylated anti-phospho-Tyr783 PLCγ1 (BS-3343-R, Bioss, Woburn, MA; 1:500) Abs. Abs were diluted in PBS plus 0.3% Triton X-100 and 1-5% normal goat or mouse serum (Jackson ImmunoResearch Laboratories, West Grove, PA, USA). Ab specificity was assessed by omitting primary Ab and, in the case of anti-CD40 mAb, by staining eyes from *Cd40*$^{-/-}$ mice.

In the case of immunoblots, membranes were probed with antibody to PLCγ1 (5690S; 1:4000), phospho-Tyr783 PLCγ1 (2821S; 1:2000), Src (2108S; 1:3000), phospho-Tyr416 Src (6943S; 1:2000), IκBα (4814P; 1:1000), phospho-Ser32 IκBα (2859S; 1:1000), JNK (9252S; 1:2000), phospho-Thr183/Tyr185 JNK (4668S; 1:3000), ERK1/2 (4695T; 1:5000), phospho-Thr202/Tyr204 ERK1/2 (4370T; 1:3000), p38 MAPK (8690T; 1:3000), phospho-Thr180/Tyr182 p38 MAPK (4511T; 1:2000), TRAF2 (4712T; 1:2000), TRAF6 (8028T; 1:2000; all from Cell Signaling Technologies, Danvers, MA) or actin (sc47778; 1:10000; Santa Cruz Biotechnologies, Santa Cruz, CA).

RNAi-mediated silencing

SMARTPool siRNAs (ON-TARGETplus) for human TRAF2, TRAF6, PLCγ1 and non-targeting siRNA were obtained from Horizon Discovery (Cambridge, UK). RNAi mediated silencing of Src was reported previously [8, 9]. Cells were transfected with siRNA (50 nM) using TransIT-X2 Dynamic Delivery System (Mirus, Madison, WI).

Flow cytometry

Human Müller cells were incubated with anti-CD40 (5C3), anti-ICAM-1 (555511, both BD Biosciences) or isotype control mAbs. Retinal cell suspensions from mice were obtained by digestion with papain (15 IU/ml)/DNAse (15 μg/ml; Worthington Biochemicals, Freehold, NJ) as described [10]. Cells were incubated with anti-CD40 (124605; BioLegend), anti-CD29 (HM β1-1, BD Biosciences, San Jose, CA) or isotype control antibodies. Flow cytometry data were acquired using an LSR II and running FACSDiva software. Expression of CD40 was analyzed on $CD29^+$ (Müller) cells.

Measurement of extracellular ATP

The ecto-ATPase inhibitor β, γ-methylene-ATP (300 μmol/l; Sigma-Aldrich, St Louis, MO) was added 15 min prior to stimulation with CD154. Extracellular ATP was quantified using an ATP bioluminescence assay kit and an ATP standard curve (Sigma-Aldrich) [11].

References

[1] Portillo J-AC, Lopez Corcino Y, Miao Y, et al. (2017) CD40 in retinal Müller cells induces $P2X_7$-dependent cytokine expression in macrophages/microglia in diabetic mice and development of early experimental diabetic retinopathy in mice. Diabetes 66: 483-493

[2] Subauste CS, Andrade RM, Wessendarp M (2007) CD40-TRAF6 and autophagy-dependent anti-microbial activity in macrophages. Autophagy 3: 245-248

[3] Hsing Y, Hostager BS, Bishop GA (1997) Characterization of CD40 signaling determinants regulating nuclear factor-kappa B activation in B lymphocytes. J Immunol 159: 4898-4906

[4] Jalukar SV, Hostager BS, Bishop GA (2000) Characterization of the roles of TNF receptor-associated factor 6 in CD40-mediated B lymphocyte effector functions. J Immunol 164: 623-630

[5] Sarao R, Dumont DJ (1998) Conditional transgene expression in endothelial cells. Transgenic Res 7: 421-427

[6] Lin W, Kemper A, McCarthy KD, et al. (2004) Interferon-γ induced medulloblastoma in the developing cerebellum. J Neurosci 24: 10074-10083

[7] Kawabe T, Naka T, Yoshida K, et al. (1994) The immune responses in CD40-deficient mice: impaired immunoglobulin class switching and germinal center formation. Immunity 1(3): 167-178

[8] Muniz-Feliciano L, Van Grol J, Portillo J-AC, et al. (2013) *Toxoplasma gondii*-induced activation of EGFR prevents autophagy protein-mediated killing of the parasite. PLoS Pathog 9: e1003809

[9] Portillo JC, Muniz-Feliciano L, Lopez Corcino Y, et al. (2017) *Toxoplasma gondii* induces FAK-Src-STAT3 signaling during infection of host cells that prevents parasite targeting by autophagy. PLoS Pathog 13(10): e1006671.

[10] Portillo J-AC, Okenka G, Kern TS, Subauste CS (2009) Identification of primary retinal cells and ex vivo identification of pro-inflammatory molecules in retinal cells using flow cytometry. Mol Vis 15: 1383-1389

[11] Blum AE, Joseph SM, Przybylski RJ, Dubyak GR (2008) Rho-family GTPases modulate Ca(2+)-dependent ATP release from astrocytes. Am J Physiol Cell Physiol 295(1): C231-241.

ESM Table 1. Average weight, blood glucose and HbA_{1c} in control and diabetic mice

Strain		Duration (m)	n	Weight (g)	Glucose (mmol/l)	HbA1c (mmol/mol)	HbA1c (%)
B6	ND	2	23	28.1 ± 0.5	7.51 ± 1.8	1	1.9 ± 0.2
	DM	2	43	26.3 ± 0.7	25 ± 0.8	57	7.4 ± 0.4
Trg-Ctr	ND	2	17	27.0 ± 0.4	7.2 ± 0.2	1	2.0 ± 0.2
	DM	2	26	24.4 ± 0.4	25.1 ± 0.9	53	7.0 ± 0.3
Trg-CD40 WT	ND	2	19	28.1 ± 0.6	7.3 ± 1	2	2.3 ± 0.3
	DM	2	34	23.8 ± 0.4	25.7 ± 0.7	54	7.1 ± 0.3
Trg-CD40 ΔT2,3	ND	2	12	28.0 ± 0.4	7.3 ± 0.2	1	2.0 ± 0.2
	DM	2	18	24.4 ± 0.7	25.5 ± 0.8	55	7.2 ± 0.3
Trg-CD40 ΔT6	ND	2	8	29.1 ± 1.1	7.4 ± 0.3	1	1.9 ± 0.2
	DM	2	8	23.4 ± 0.7	26.1 ± 0.8	55	7.2 ± 0.3
B6	ND	8	6	36 ± 2.4	7.7 ± 0.5	16	3.6 ± 0.1
	DM	8	8	27 ± 1.3	27.5 ± 1..4	100	11.3 ± 0.9
Trg-Ctr	ND	8	6	34.6 ± 2.5	7.6 ± 0.4	15	3.5 ± 0.1
	DM	8	6	26 ± 0.5	27.4 ± 1.5	98	11.1 ± 0.7
Trg-CD40 WT	ND	8	6	35 ± 2.3	7.5 ± 0.6	15	3.5 ± 0.1
	DM	8	8	25.5 ± 0.5	27.0 ± 2.0	104	11.7 ± 1
Trg-CD40 ΔT2,3	ND	8	6	34.6 ± 2.5	7.2 ± 0.4	15	3.5 ± 0.1
	DM	8	7	25 ± 2.7	27.9 ± 1.5	107	11.9 ± 0.9

ND, Non-diabetic; DM, Diabetic

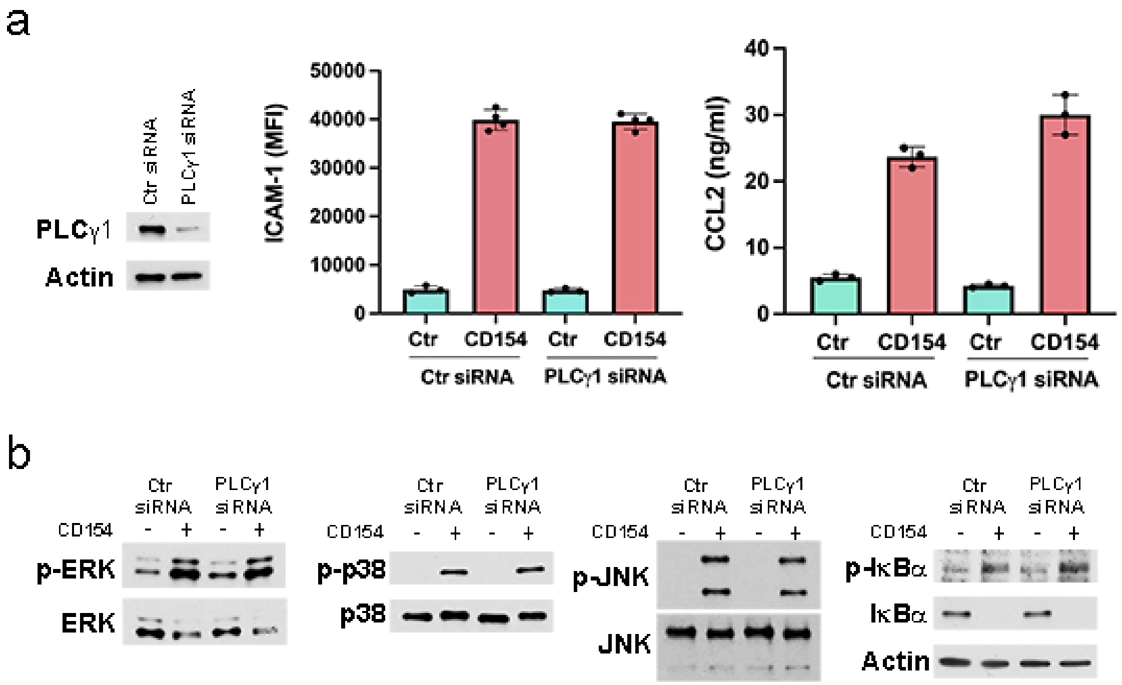

ESM Fig. 1. PLCγ1 is not required for upregulation ICAM-1 and CCL2 in Müller cells as well as for activation of signaling molecules that drive expression of these pro-inflammatory molecules. Human Müller cells were transfected with control or PLCγ1 siRNA. *a*, Immunoblot confirmed knockdown of PLCγ1. Müller cells were stimulated with or without CD154. ICAM-1 expression was examined by flow cytometry while CCL2 production was examined by ELISA (both at 24 h). *b*, Phosphorylation of ERK1/2, JNK, p38 MAPK and NFκB were examined at 30 min. Representative results of 2-3 different experiments.

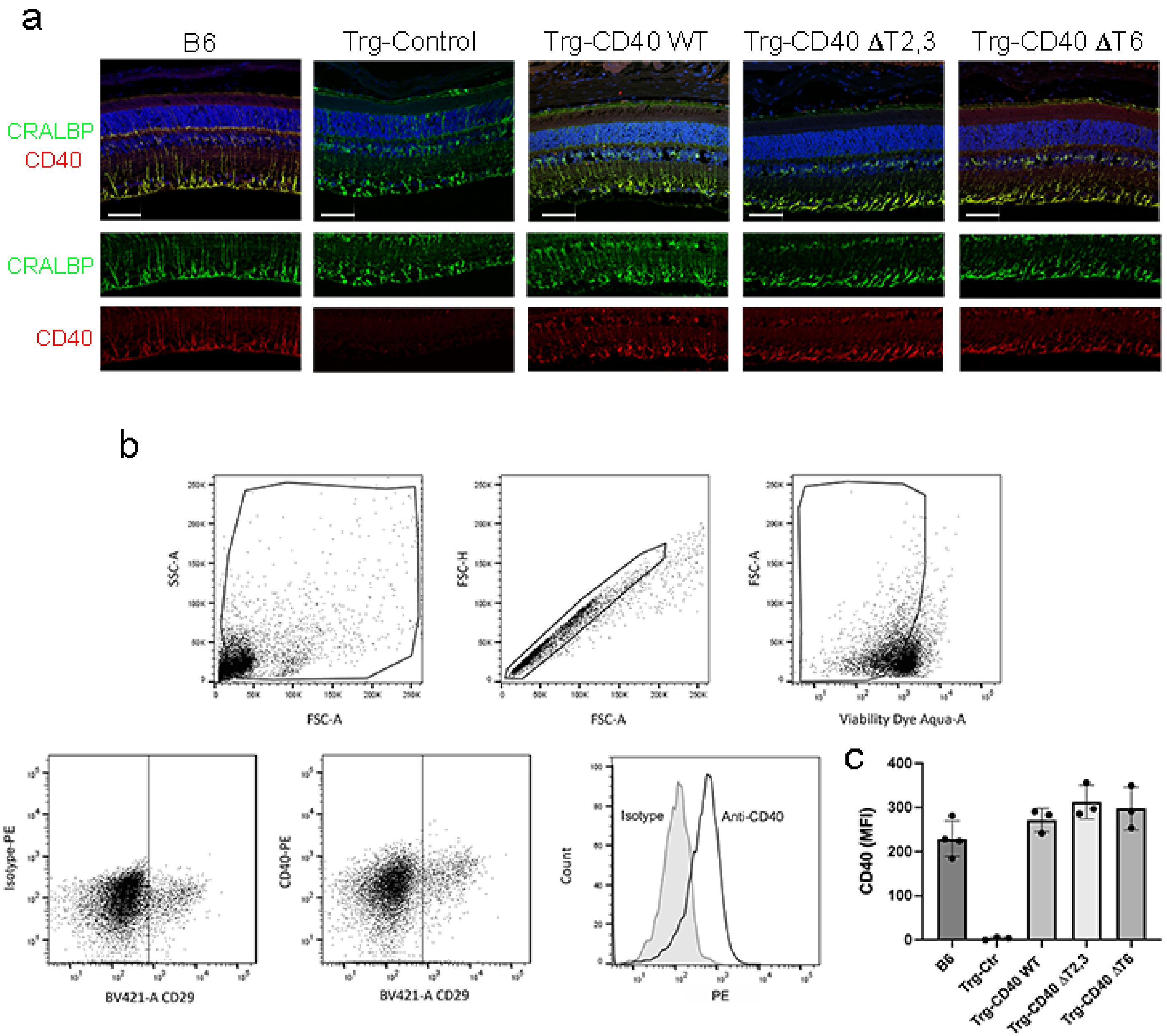

ESM Fig. 2. Transgenic mice with expression of CD40 WT or CD40 with mutations in the TRAF binding sites. *a*, CD40 expression in Müller cells (CRALBP+) from B6, single transgenic (Trg-Ctr) or double transgenic mice that express CD40 WT (Trg-CD40 WT), CD40 ΔT2,3 (Trg- CD40 ΔT2,3) or CD40 ΔT6 (Trg-CD40 ΔT6). Scale bar, 50 µm. *b*, CD40 expression on Müller cells (CD29+) isolated from the retina as assessed by flow cytometry. Dot plot show gating strategy. CD40 was analyzed on live cells (did not stain with Aqua LIVE/DEAD kit) that were CD29+ (Müller cells). Dot plots and histogram shows representative CD40 expression in a B6 mouse. c, Graph bars shows mean + SEM of CD40 mean fluorescence intensity (3-4 mice per group).

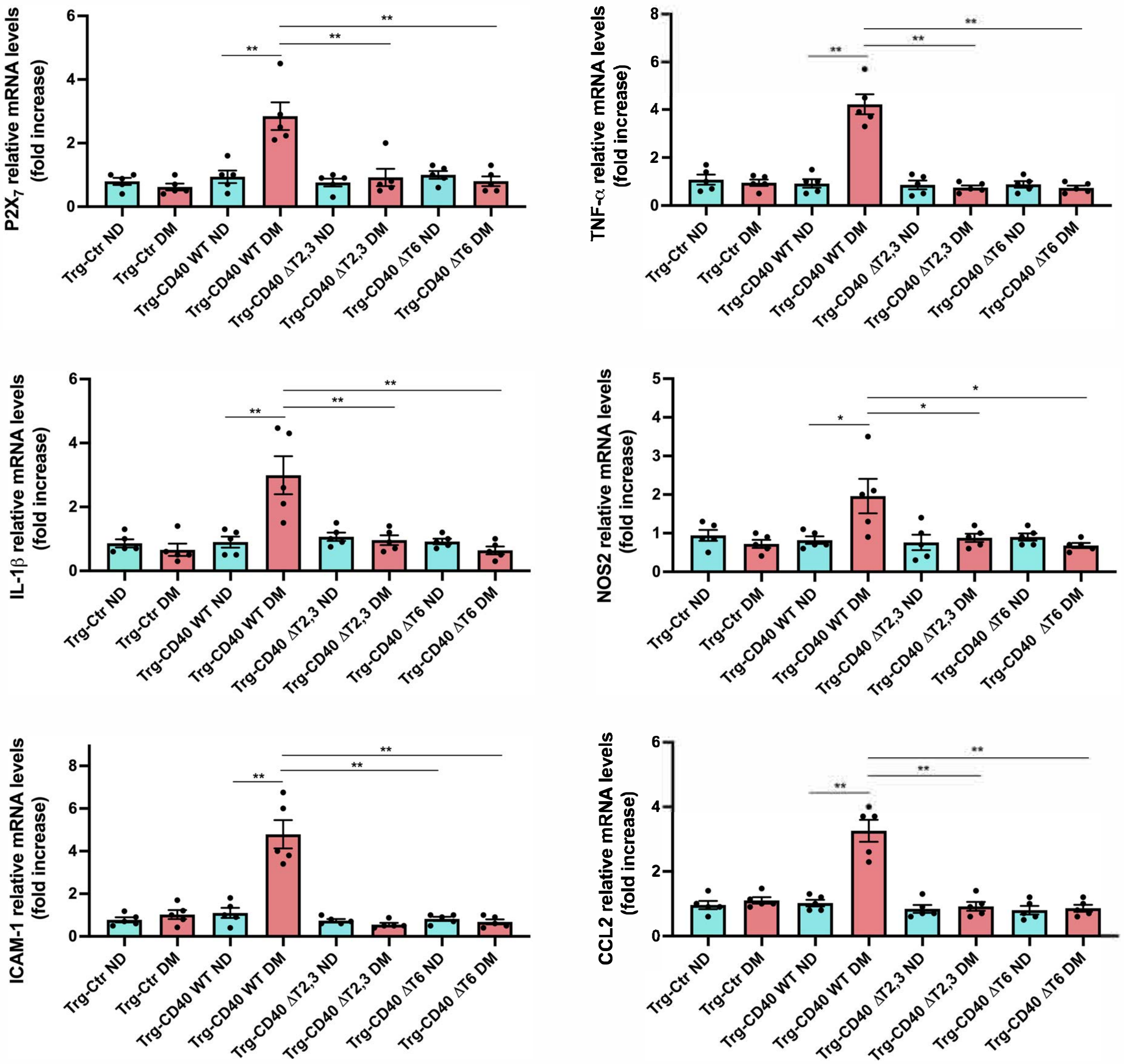

ESM Figure 3. Expression of CD40 ΔT6 in Müller cells from diabetic mice prevents upregulation of *P2X*$_7$, *Tnf-*α, *Il-1*β, *Nos2*, *Icam-1* and *CCL2* mRNA in the retina. At 2 m of diabetes, mRNA levels were assessed by real time quantitative PCR using 18S rRNA as internal control. One non-diabetic B6 mouse was given an arbitrary value of 1 and data are expressed as fold-increase compared to this animal. Horizontal bars represent mean +/- SEM. 5 animals per group. * $P < 0.05$, ** $P < 0.01$ by ANOVA.
